# Herpes Vegetans in a Patient With Acquired Immunodeficiency Syndrome (AIDS) in the Setting of Immune Reconstitution Inflammatory Syndrome

**DOI:** 10.7759/cureus.67219

**Published:** 2024-08-19

**Authors:** Angelique Ruml, Ibeth Caceres, Anna Catinis, Theodore Rosen

**Affiliations:** 1 Department of Dermatology, Baylor College of Medicine, Houston, USA; 2 Department of Dermatology, Michael E. DeBakey Veterans Affairs Medical Center, Baylor College of Medicine, Houston, USA

**Keywords:** immune reconstitution inflammatory syndrome, iris, aids, hypertrophic herpes simplex, herpes vegetans, case report

## Abstract

Herpes simplex virus (HSV) infections classically present as a vesicular eruption on an erythematous base; however, viral infections may present much differently in the setting of immune deficiency. Herpes vegetans is an atypical presentation of HSV that occurs in immunocompromised patients, typically those with human immunodeficiency virus infection and acquired immunodeficiency syndrome (AIDS). Herpes vegetans is characterized by hyperkeratotic, exophytic, and, sometimes, ulcerated nodules, often with a chronic and persistent course. Herein, we present an interesting example of biopsy-confirmed anogenital herpes vegetans in a 61-year-old male with AIDS in the setting of immune reconstitution inflammatory syndrome, an association that is less frequently described. This case serves as an important reminder to consider atypical presentations of infectious disease when examining immunocompromised patients, as prompt diagnosis and treatment are essential in this population.

## Introduction

Herpes vegetans, or hypertrophic herpes simplex, is a rare, atypical cutaneous variant of the herpes simplex virus (HSV), presenting almost exclusively in immunocompromised individuals [1]. Based on serologic studies, the national seroprevalence of HSV-1 and HSV-2 is 47.8% and 11.9%, respectively [2]. HSV-2 is primarily responsible for herpes vegetans [1]. Annually, there are an estimated 570,000 new genital herpes infections, with HSV-2 infection rates being disproportionately higher among non-Hispanic Black patients (34.6%) compared to non-Hispanic White patients (8.1%) [2].

Clinically, the herpes vegetans variant presents as hyperkeratotic, verrucous plaques and papulonodules that may ulcerate and erode, often resembling malignant neoplasms [3]. Lesions are classically present in the anogenital region. However, involvement of disparate sites such as the oral mucosa and eyelid has been reported [4-7]. Understanding the pathophysiology of HSV infection justifies the atypical clinical presentation of HSV in immunocompromised individuals. HSV predominately affects epithelial and neuronal cells. Initial infection involves epithelial replication, classically producing a vesicle on an erythematous base. The virus subsequently travels to the dorsal root ganglia by retrograde axonal flow, establishing latency. Upon reactivation, the virus spreads distally from the ganglion to initiate new cutaneous and/or mucosal lesions [8]. Toll-like receptors (TLRs), proteins on the surface of immune cells that can recognize microbial pathogens, are critical to the first line of innate immune defense against HSV, as they help prime CD8+ T cells. CD8+ T cells recognize antigens on virally infected cells, triggering the release of cytolytic molecules leading to cell death​ [9]. HSV-specific memory CD8+ T cells in affected dorsal root ganglia help control the infection and prevent recurrences [9]. However, immunocompromised patients have an inadequate response to TLR-9 in their dendritic cells, leading to decreased interferon-alpha production [10]. Interferon-alpha is a known physiologic regulator of epidermal growth in vivo, and its downregulation, along with the myriad of other inflammatory cytokines that are typically upregulated in the setting of immune reconstitution inflammatory syndrome (IRIS), is thought to contribute to the development of exophytic lesions of herpes vegetans seen in this patient population [11,12].

IRIS, an exaggerated inflammatory response following treatment initiation in immunocompromised individuals, is commonly observed in those with HIV undergoing antiretroviral therapy (ART) [13]. This hyperinflammatory response in immunosuppressed individuals occurs secondary to the regained capacity to mount an immune response [13]. IRIS may manifest as either the unmasking or paradoxical form. The unmasking form of IRIS involves worsening a previously unrecognized infection in the ART setting, as observed in this case [13]. In contrast, the paradoxical form consists of worsening a preexisting infection [13].

Histologically, these vegetative lesions are characterized by pseudoepitheliomatous hyperplasia (i.e., a reactive epithelial proliferation) of the epidermis, multinucleated keratinocytes containing molded nuclei, and intranuclear viral inclusions [14]. A dense mixed dermal infiltrate of lymphocytes, histiocytes, plasma cells, and eosinophils may be found beneath the ulcer base [3]. We thus present an uncommon presentation of a common infection in the setting of severe immune compromise and IRIS.

## Case presentation

**Figure 1:**
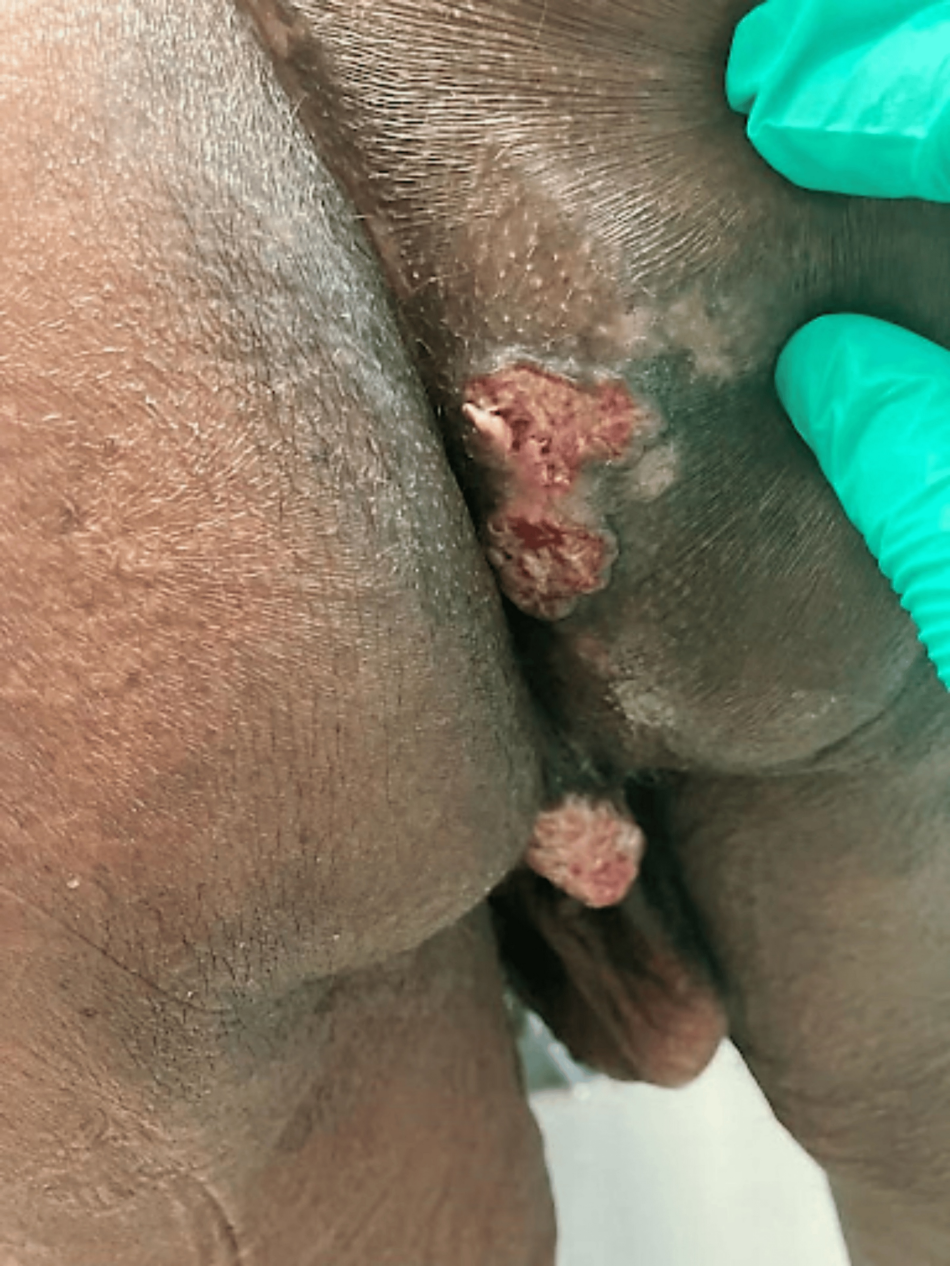
Well-circumscribed ulcerated plaques with rolled borders on the right buttock and a large fungating, friable tumor posterior to the scrotum

A 61-year-old African American male with human immunodeficiency virus (HIV) infection diagnosed in 2003 and acquired immunodeficiency syndrome (AIDS) (CD4 of 171 cells/mm^3^ and viral load of 187 copies/mm^3^) with a history of poor adherence to ART presented to the Veterans Affairs Dermatology clinic with ulcerated plaques on his buttocks and a tender fungating mass with milky drainage located posterior to his scrotum for three months (Figure 1). He had been taking his ART more regularly recently, resulting in improved CD4 count and viral load. He denied weight loss, fever, chills, or night sweats. He had no recent travel or contact with ill individuals. He had no similar lesions elsewhere. Infectious disease workup, including screening for gonorrhea, chlamydia, tuberculosis, and syphilis, was unremarkable. The differential diagnosis at this time was vast. It included condyloma acuminata (genital warts), condyloma lata of secondary syphilis, Buschke-Lowenstein tumor, verrucous or invasive squamous cell carcinoma, pemphigus vegetans, endemic fungal or atypical mycobacterial infection, pyoderma gangrenosum, and late gummatous syphilis.

**Figure 2:**
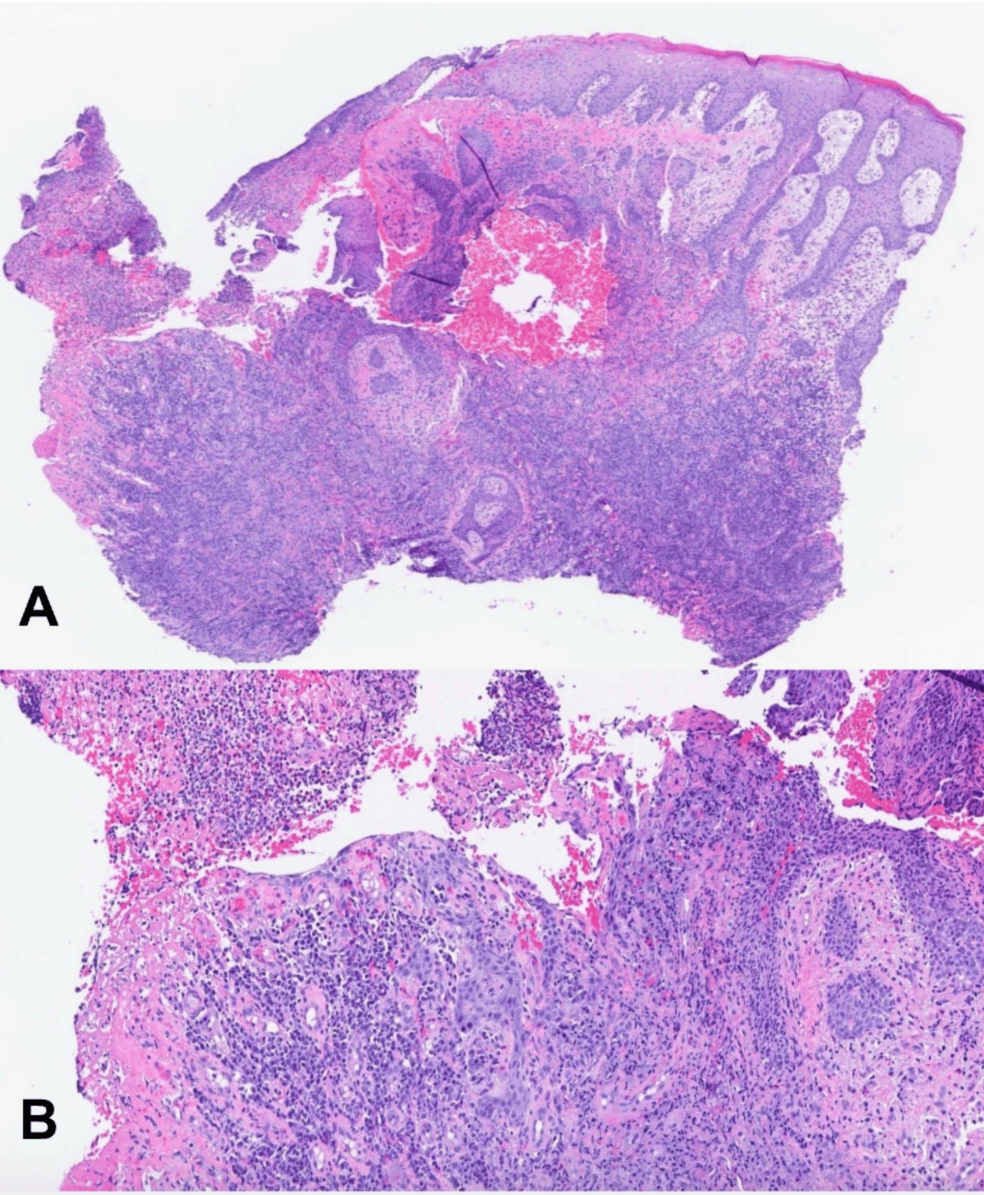
(A) Hematoxylin-eosin stain, 20x, revealing chronic and acute inflammation consisting of plasma cells, histiocytes, neutrophils, lymphocytes, and massive pseudoepitheliomatous epidermal hyperplasia. (B)​ Hematoxylin-eosin stain, 100x, showing an ulcer with chronic and acute mixed inflammation consisting of plasma cells, histiocytes, neutrophils, and lymphocytes

**Figure 3:**
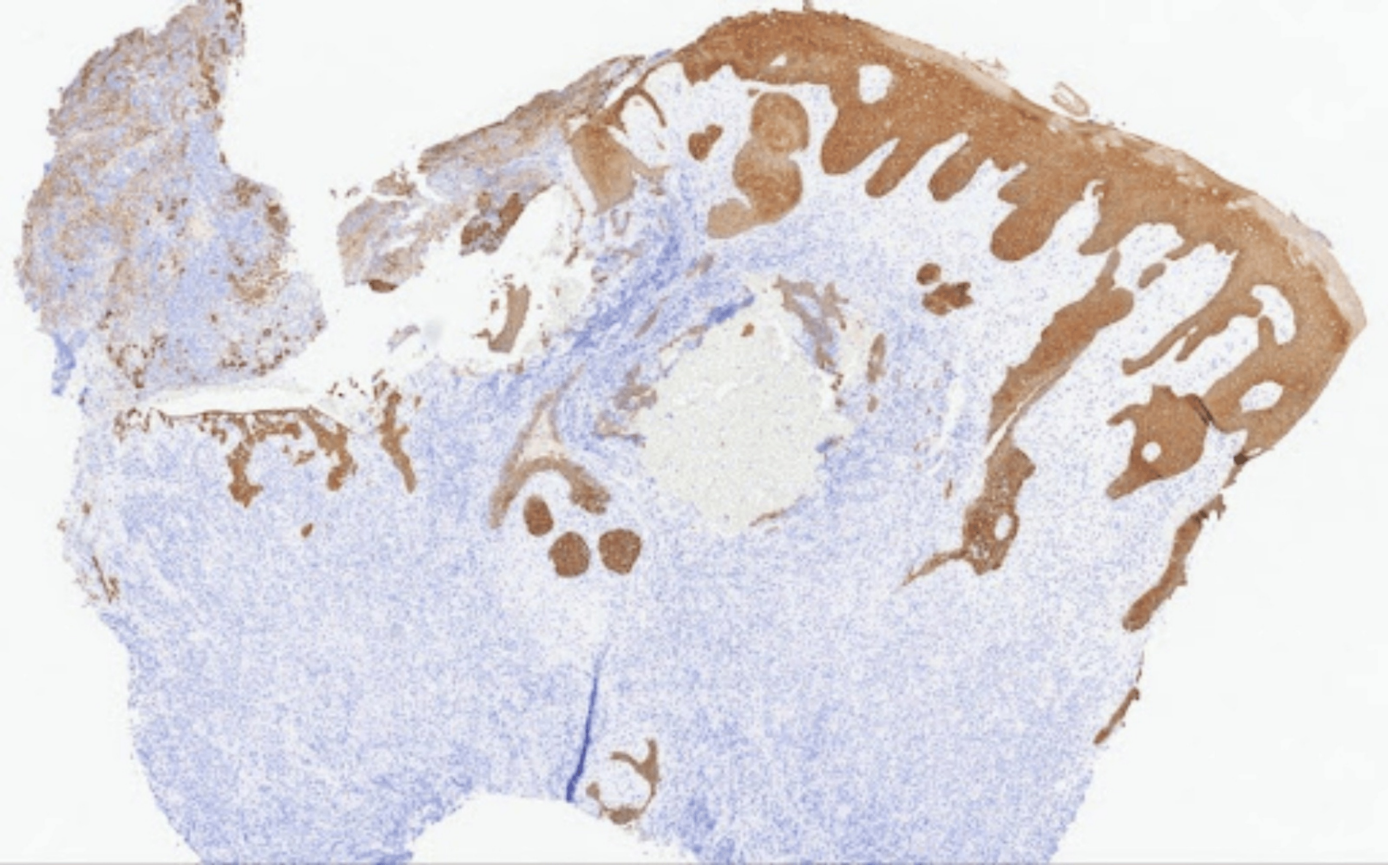
Pancytokeratin stain, 20x, highlighting the epidermis and entrapped squamous epithelium in the ulcer

**Figure 4:**
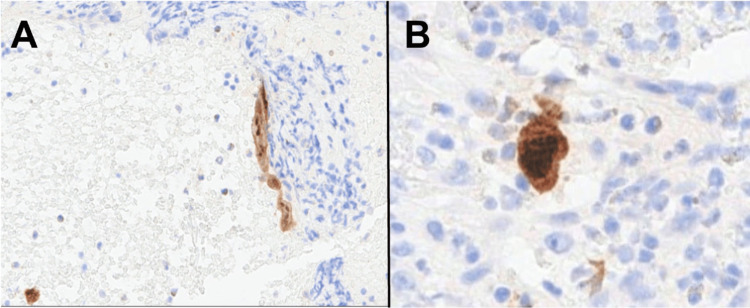
HSV-1/2 immunostain highlighting rare, infected cells at (A) 200x and (B) 400x HSV: herpes simplex virus

Punch biopsies of the posterior scrotal and gluteal lesions were obtained and sent for histology and culture. Histopathology revealed intense acute and chronic inflammation, including abundant plasma cells, histiocytes, neutrophils, and lymphocytes with pseudoepitheliomatous epidermal hyperplasia (Figures 2A, 2B). Given our leading suspected diagnoses of malignancy and infection, several stains were performed to aid in narrowing our differential. Pancytokeratin stain was not suggestive of squamous cell carcinoma (Figure 3). Spirochete (*Treponema pallidum*) immunohistochemistry, acid-fast, and Grocott methenamine silver stains were negative for causative organisms. HSV-1 and HSV-2 immunohistochemical stains were focally positive, confirming the diagnosis of HSV infection (Figures 4A, 4B). The patient was subsequently started on a three-week course of oral valacyclovir therapy 500 mg twice daily with a complete response. At five months after treatment, the patient reported maintained clearance without recurrence.

## Discussion

HSV, a common sexually transmitted infection among HIV-positive patients, presents variably in the immunodeficient patient, ranging in morphology from chronic ulcerations to rapidly growing exophytic lesions [15]. Therefore, it is imperative to consider atypical presentations of this ubiquitous infection in this select patient population [4,15]. This is particularly important for immunocompromised patients with skin of color, especially non-Hispanic Black patients, who are disproportionately affected by genital herpes infections [2].

When the clinical impression is unreliable, diagnosis depends on a comprehensive assessment integrating the clinical, histological, immunopathological, microbiological, and serological findings. The differential diagnosis of these ulcerated, vegetative plaques in the anogenital region of an immunocompromised patient was vast. To name a few, fungal or atypical mycobacterial infection​ was unlikely in our patient given the negative tuberculosis screening, acid-fast stain, and silver stain; squamous cell carcinoma was unlikely without evidence of abnormal keratin proliferation; gummatous syphilis was unlikely in the setting of negative syphilis screening and spirochete staining. Tissue samples for histology and culture are necessary for workup, as superficial viral cultures (as might be collected by swabbing the lesion) are often negative, and biopsies may not reveal typical histopathologic features such as viral inclusions due to impaired cell-mediated immunity [16,17]. Consistent with preexisting literature, this case illustrates the benefit of early biopsy and tissue culture when presented with a vegetative mass or ulcerations in the anogenital area in an immunocompromised host to prevent diagnostic and treatment delays. For example, one study of genital herpes infections in adults found that characteristic ulcerations of the external genitalia were present in only two-thirds of the individuals with positive HSV cultures, evidence of the problem of underdiagnosis of these infections in the setting of increasing seroprevalence [18].

The degree of immunosuppression, characterized by CD4 count and viral load, is also critical when evaluating patients with HIV/AIDS for infection. Our patient began taking ART more consistently in the months preceding symptom onset, suggesting his vegetative plaques were likely secondary to, or exacerbated by, IRIS, a potential complication of the use of ART. While the exact mechanism is not fully understood, IRIS is a dysregulated, hyperinflammatory response that occurs with immune reconstitution, especially in the setting of opportunistic infections. It contributes to the development of herpes vegetans, specifically, as HIV itself tends to infect dermal dendritic cells, which respond by producing tumor necrosis factor and interleukin-6 via TLRs 7 and 9 [12]. These cytokines, extraordinarily upregulated in the setting of IRIS, create an antiapoptotic environment that favors the proliferation of the epidermis and the eventual hyperkeratotic and verrucous appearance of the skin [12].

The incidence of IRIS ranges from 25% to 30% of patients with HIV on ART, usually occurring within the first six months of treatment [13]. In addition to increasing the risk of resistance to ART and impairing quality of life, IRIS can also contribute to poor adherence to ART, perpetrating a harmful cycle of uncontrolled infection. IRIS is associated with significant morbidity and mortality in HIV/AIDS patients, and prompt recognition of this condition is necessary for timely management and improved outcomes [13]. Management of IRIS should focus on symptom control, start antimicrobial agents to target the underlying opportunistic infection, and continue ART unless there is evidence of severe drug-related toxicity or IRIS affecting the central nervous system [13].

Future studies are needed to determine optimal treatment in the setting of IRIS. However, the mainstay of treatment for herpes vegetans includes initiating appropriate antiviral medications concurrently with the institution of ART. First-line antivirals include guanosine analogs such as acyclovir, famciclovir, and valacyclovir, the latter of which has greater bioavailability [15]. Other options include imiquimod, foscarnet, or cidofovir,​ which are primarily considered in viral resistance due to mutations in thymidine kinase or, less commonly, in DNA polymerase [15]. One review found that only 30% of herpes vegetans cases studied were susceptible to acyclovir, with the remaining cases requiring management with surgery or alternative systemic agents [15].

There have also been reports of topical imiquimod as both an effective primary or adjunctive therapy for vegetative HSV infection. One systematic review that searched for clinical studies reporting the complete cure rate and the time to complete response in patients >18 years old with hypertrophic HSV infection found that topical 5% imiquimod therapy achieved a complete cure rate of 88.1% with a median time to complete response of 60 days [19]. This outcome was better than using thymidine kinase-dependent antivirals alone, which was reported to have a complete cure rate of 62.5% [19]. Imiquimod acts as an upregulating modulator of T cell-mediated adaptive immune function as opposed to a direct antiviral agent. Imiquimod should be kept in mind, particularly in the setting of acyclovir resistance or unresponsiveness [15,20].

Setting patient expectations before treatment initiation is beneficial, as lesions can be relapsing or persistent [15]. While primary skin infections often recur and are challenging to treat in HIV-positive patients, we can expect them to serve as an increasing health burden as patients live longer, making knowledge of management options increasingly relevant [16].

## Conclusions

HSV infections are common, classically presenting as readily identifiable grouped vesicles on an erythematous base. However, immunosuppressed individuals may present with rare, atypical morphologies, such as the verrucous, proliferative lesions characteristic of herpes vegetans. As the differential diagnosis with such lesions in an immunocompromised host is broad, this case was an important reminder to our team to maintain a high index of suspicion for infectious etiologies when evaluating immunocompromised patients with new neoplasms, especially in the context of possible immune reconstitution. Such clinical vigilance is warranted to ensure prompt diagnosis and treatment, and interdisciplinary follow-up can be considered (i.e., infectious disease) to optimize patient outcomes.
